# Accessing the exceptional points of parity-time symmetric acoustics

**DOI:** 10.1038/ncomms11110

**Published:** 2016-03-30

**Authors:** Chengzhi Shi, Marc Dubois, Yun Chen, Lei Cheng, Hamidreza Ramezani, Yuan Wang, Xiang Zhang

**Affiliations:** 1 NSF Nano-scale Science and Engineering Center (NSEC), University of California, Berkeley, 3112 Etcheverry Hall, Berkeley, California 94720, USA; 2 State Key Laboratory of ASIC and System, Department of Microelectronics, Fudan University, 201203 Shanghai, China; 3 Materials Science Division, Lawrence Berkeley National Laboratory, 1 Cyclotron Road, Berkeley, California 94720, USA; 4 Department of Physics, King Abdulaziz University, Jeddah 21589, Saudi Arabia

## Abstract

Parity-time (PT) symmetric systems experience phase transition between PT exact and broken phases at exceptional point. These PT phase transitions contribute significantly to the design of single mode lasers, coherent perfect absorbers, isolators, and diodes. However, such exceptional points are extremely difficult to access in practice because of the dispersive behaviour of most loss and gain materials required in PT symmetric systems. Here we introduce a method to systematically tame these exceptional points and control PT phases. Our experimental demonstration hinges on an active acoustic element that realizes a complex-valued potential and simultaneously controls the multiple interference in the structure. The manipulation of exceptional points offers new routes to broaden applications for PT symmetric physics in acoustics, optics, microwaves and electronics, which are essential for sensing, communication and imaging.

## Abstract (web summary)

Parity-time phase transition and exceptional points contribute to the design of many components, but are hard to access due to the dispersive nature of the required materials. Here, the authors introduce a method to systematically control acoustic parity-time phase transition through an active element.

## Graphical abstract

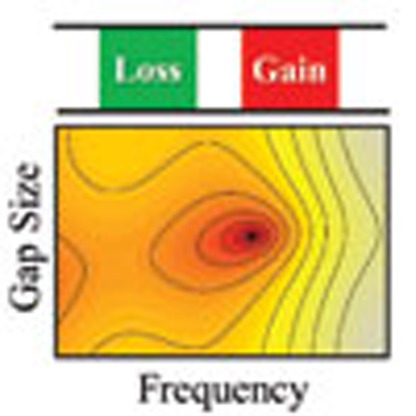

Non-reciprocal wave transport is important for key applications such as isolators^1^,^2^,^3^, diodes^4^,^5^, and rectifiers^6^,^7^. However, these schemes rely on high-order harmonic generation, mode conversion or angular-dependent bandgap in order to realize asymmetric transmission. Recent progress on parity-time (PT) symmetric systems provides a new paradigm to realize asymmetric transport without frequency or mode conversion^8^,^9^,^10^,^11^,^12^,^13^,^14^,^15^,^16^,^17^,^18^,^19^,^20^. Only at specific frequencies called exceptional points, complex interference in PT symmetric systems results in unidirectional reflection that is crucial for applications^9^,^10^,^21^. PT symmetric systems are invariant under parity and time-reversal operations. Therefore, they obey the following energy conservation relation:


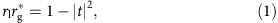


where *t* is the transmission coefficient, *r*_1_ is the reflection coefficient from the loss side and *r*_g_ is the reflection coefficient from the gain side of the system^16^,^21^. In PT symmetric systems, phase transition, where the system Hamiltonian switches between real and complex spectrum^22^,^23^, provides a novel approach to realize asymmetric transport. This phase transition has been intensely studied in electronics^24^,^25^,^26^, optics^8^,^9^,^10^,^11^,^12^,^13^,^14^,^18^,^19^,^20^,^27^,^28^,^29^, and acoustics^16^,^17^. The right-hand side sign of equation (1) determines the PT phase of the system: positive for PT exact phase and negative for PT broken phase^16^,^21^. When the system undergoes phase transition, transmission is unity, |*t*|=1, corresponding to the existence of exceptional points. Therefore, one of the reflections must vanish at these exceptional points, typically resulting in unidirectional transparency. The PT symmetric condition requires the distribution of complex refractive index to be a Hermitian function in the transverse direction such that *n* (*x*)=n*(−*x*) (refs 8, 10). This distribution is achieved by precisely balancing loss and gain materials. However, PT symmetric systems require an exact balance of loss and gain in the medium, and the dispersive behaviour of most loss and gain materials prevents this condition to be fulfilled over a broad frequency range. Hence, it is challenging to access the exceptional points in practice.

Here, we present a general method to approach the exceptional points within a large frequency range by tuning an acoustic gain medium that satisfies the PT symmetric condition and simultaneously controls the scattering process. We experimentally demonstrate the ability to reverse the direction of the acoustic transparency at any given frequency. Such control of directional transparency opens new routes for wave physics including acoustics and electromagnetics, and important applications in sensing, communication and imaging.

## Results

### Tailoring the exceptional points of PT symmetric structure

**Figure 1:**
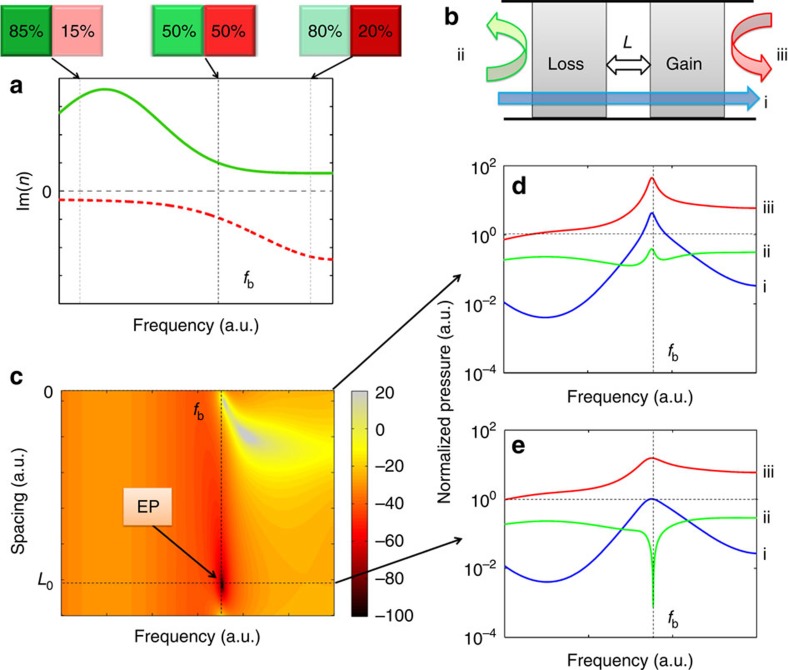
Accessing the exceptional point of acoustic PT system by tuning the spacing between loss and gain materials. (**a**) Imaginary parts of the refractive indices of loss and gain materials are typically dispersive, thus the PT symmetric condition could only be satisfied at single frequency *f*_b_ where the loss and gain materials are exactly balanced. (**b**) Loss and gain materials assembled with spacing. (**c**) Amplitude of the reflection from the loss side as a function of frequency and the spacing between the loss and gain materials. Exceptional point (EP) occurs at frequency *f*_b_ and a specific spacing *L*_0_ when the reflection vanishes. (**d**) The normalized transmissions (blue, i), reflections from the loss (green, ii), and from the gain (red, iii) in logarithmic scale without spacing, no exceptional point observed. (**e**) Similar representation to **d** with spacing *L*_0_, an exceptional point observed at *f*_b_.

Figure 1 illustrates the control of exceptional points for typical dispersive loss and gain materials, the imaginary parts of their indices are balanced at a single frequency *f*_b_. When there is no spacing between the loss and gain materials (Fig. 1d), one can observe reflections from both sides, but cannot access exceptional points. Exceptional points and unidirectional transparency are the results of complex interference inside the PT symmetric structure. This scattering process can be controlled by introducing a gap corresponding to a phase delay between the gain and loss cells (Fig. 1b). Analytic calculations based on transfer-matrix formalism are used to scan the response of the structure as a function of spacing and frequency. The reflection from the loss side (Fig. 1c) presents a sharp dip for a specific spacing *L*_0_ at frequency *f*_b_, where the multi-reflection interfere destructively. In this case, one can access an exceptional point where the reflection from the loss side vanishes while achieving total transmission and nonzero reflection from the gain side, making the system to be unidirectional transparent (Fig. 1e).

### Experimental demonstration of unidirectional transparency

**Figure 2:**
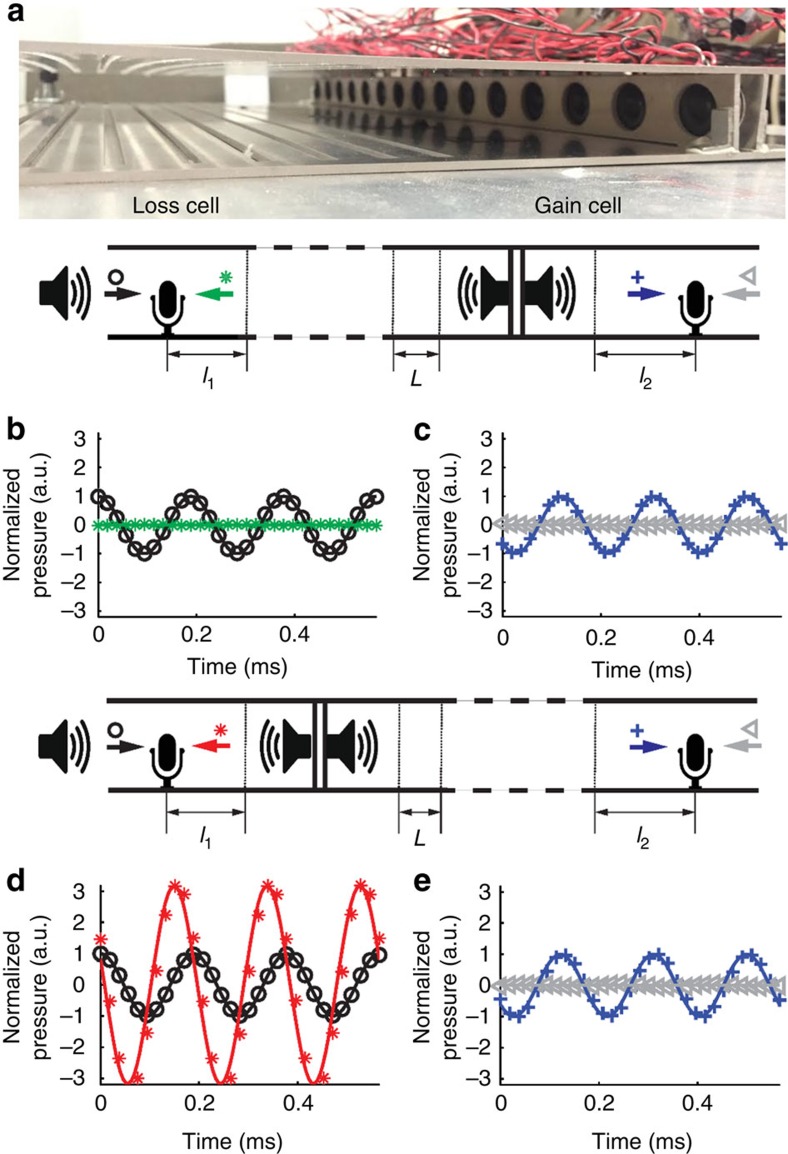
Experiment demonstration of unidirectional transparency of the PT symmetric system at 5.3 kHz. (**a**) The photo of an experimental sample including loss and gain units. (**b**,**c**) The calculated (solid curves) and measured (marked dots) transmissions and reflections when the incident wave is coming from the loss side. (**d**,**e**) Similar representation to **b** and **c**) when the incident wave is from the gain side. Black, green, red, blue and grey colours denote the incidence, reflection from the loss side, the reflection from the gain side, the transmission and the reflection from the end of the waveguide, respectively. All results have been normalized with the amplitude of incidence. The two calibrated unidirectional microphones are mounted at *l*_1_=15.5 cm and *l*_2_=13 cm away from the boundaries of our PT symmetric materials. The spacing between the loss and gain materials is *L*=1.24 cm. No reflection is observed from the loss side (green curve and dots in **b**), ∼330% reflection is observed from the gain side (red curve and dots in **d**), and total transmissions (|*t*|=1) have been observed on both sides, resulting in unidirectional transparency from the loss side.

**Figure 3:**
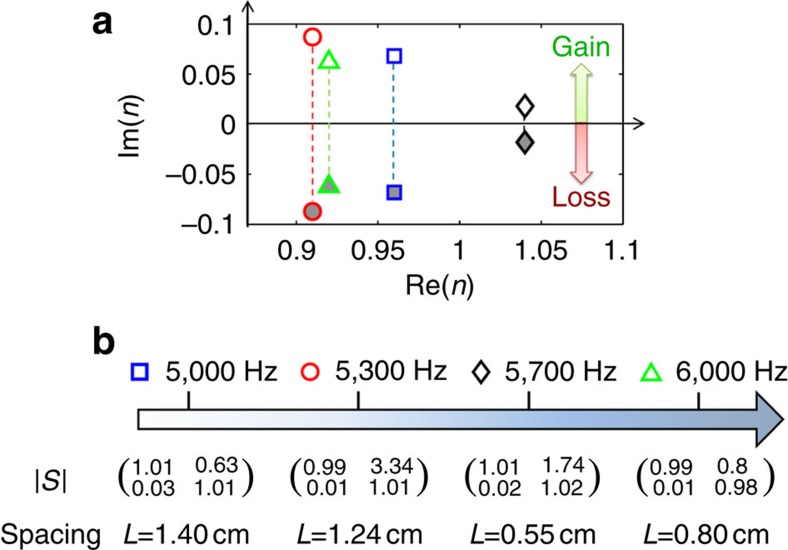
Taming exceptional points at different frequencies. (**a**) The measured complex-valued refractive indices of the loss material formed by the leaky waveguide at 5, 5.3, 5.7 and 6 kHz (open marks). The active gain material is tuned to have the refractive indices that are complex conjugate of those of the loss material at the corresponding frequencies (filled marks). (**b**) The measured amplitudes of the scattering matrices for the controlled exceptional points at these four frequencies with the appropriately tuned spacing between the loss and gain materials. The reflections from the loss side almost vanish and total transmissions are observed for all of the frequencies. The reflections from the gain side are nonzero.

Recent development in PT symmetric acoustics promises new applications such as asymmetric cloaking and sensing^16^,^17^. The control of exceptional points is crucial for the design of such devices. The realization of PT symmetric acoustics relies on the combination of acoustic loss and gain materials. It has been shown that active elements can be tuned to obtain artificial materials with desired effective acoustic parameters. This was demonstrated in the modulation of the real part of the acoustic parameters^30^. In this work, we explore the acoustic parameters in the complex domain to exploit non-Hermitian physics and the control of exceptional points. In our experiments, coherent acoustic sources are used to generate a gain response that balances a passive loss material and satisfies the PT symmetric condition. Our acoustic prototype consists of one loss cell, where multiple slits are carved in a waveguide to form a leaky region, and one gain unit, formed by two acoustic source arrays paired with upstream and downstream highly directive sensors (Fig. 2a). We first obtain the scattering matrix of the loss part at 5.3 kHz by measuring the amplitude and phase of transmission and reflection without the gain unit. This frequency corresponds to a local maximum of the loss parameters, resulting in high contrast between the reflections of the PT symmetric system (Fig. 3a). A parameter extraction algorithm is used to retrieve the effective parameters of the loss material from the measured scattering matrix at 5.3 kHz (ref. 31). The calculated mass density is 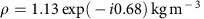 and bulk modulus is 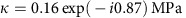 (refractive index is 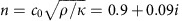 with *c*_0_=343 ms^−1^). Effective parameters of the gain medium are obtained by complex conjugation, and the same algorithm can be reversed to calculate the gain scattering matrix. By tuning the two acoustic source arrays to match this scattering matrix, the PT symmetric condition can be satisfied at 5.3 kHz. As discussed previously, exceptional point occurrences are dictated by the multiple interferences within the structure. In our experiment, these interferences are controlled by the spacing between the loss and gain materials. Analytical calculation at 5.3 kHz reveals that 1.24-cm spacing results in unidirectional transparency from the loss side. The loss region and the balanced gain unit are assembled in a waveguide with the correct spacing. The scattering matrix of the entire system is measured by two calibrated microphone pairs


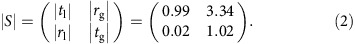


The scattering matrices of the loss and balanced gain units are given in Supplementary Note 1. The experimental results confirm the unidirectional transparency from the loss side, which agree well with the analytical predictions (Fig. 2b–e).

### Accessing exceptional points at multiple frequencies

One major benefit of using active gain material is that the PT symmetric condition can be satisfied over a large frequency band, regardless of the loss material dispersion. We demonstrate that unidirectional transparency is achieved by controlling exceptional points for different frequencies within the range of 5–6 kHz. The scattering matrices of the leaky waveguide are measured at four frequencies and the retrieved refractive indices confirm the dispersive behaviour of our loss part (Fig. 3a). The complex conjugates of these indices are used to calculate the scattering matrices of the gain material at each frequency. In order to observe unidirectional transparency at these frequencies, different spacing are required in the system. The calculated spacing distances and the measured scattering matrices are listed in Fig. 3b. By applying opposite phase shifts to the two acoustic source arrays, we introduce an effective spatial offset between the gain medium and these emitting arrays. The phase shifts allow us to set the effective spacing between the loss and gain materials in a fixed structure for each frequency (Supplementary Note 2). For all measurements, total transmissions through the PT symmetric system can be observed within 2% error, the reflections from the loss side vanish (<3%) and the reflections from the gain side are nonzero. It is important to point out that both the loss and gain units are working in linear conditions. Thus, the system will preserve the unidirectional reflection when input signals are linear combinations of the demonstrated frequencies, promising time-dependent signal applications.

### Reversing the orientation of unidirectional transparency

**Figure 4:**
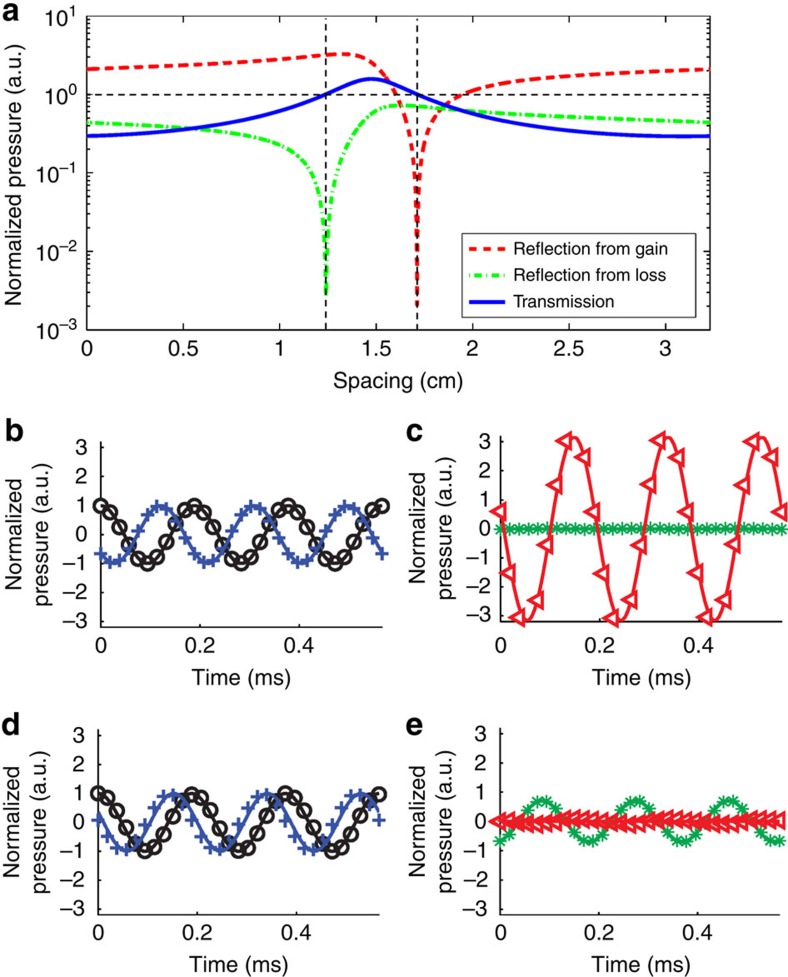
Reversing the unidirectional transparency at 5.3 kHz. (**a**) Calculated transmission (blue) and reflections from the gain side (orange) and the loss side (red) with varying spacing between the loss and gain materials from zero to half wavelength. Two exceptional points are observed when the spacing is 1.24 and 1.71 cm, respectively. (**b**,**c**) Transmission and reflections from the gain and loss sides associated with 1.24-cm spacing. Black, blue, red and green colours denote the incidence, transmission, reflection from the gain side and reflection from the loss side, respectively. Solid curves are calculated values, and marked dots are from measurements. The same as in Fig. 2, unidirectional transparency is observed from the loss side. (**d**,**e**) Similar representation to **b** and **c**, but with 1.71-cm spacing. The direction of unidirectional transparency is reversed. Nearly zero reflection from gain side and 70% reflection from the loss side are observed.

Previous studies in optics and acoustics only demonstrate unidirectional transparency from the loss side^10^,^17^, whereas equation (1) reveals that two types of exceptional points exist depending on the side where reflection is cancelled. Here we demonstrate that our method can control the orientation of unidirectional transparency at the same working frequency, which is useful for applications such as logical acoustics. Figure 4a presents the transmission and reflection coefficients of the PT symmetric system as functions of spacing. These coefficients are periodic functions with period *λ*/2. Within this scope, two exceptional points are observed. The same as shown in Fig. 2, reflection from the loss side vanishes with 1.24-cm spacing. The other exceptional point occurs at 1.71-cm spacing, where the reflection from the gain side is cancelled. Figure 4b–e shows the transmissions and reflections normalized with their inputs for these two different gaps. In both cases, we can observe total transmissions of waves. Once we tune the spacing to be 1.71 cm, 70% reflection from the loss side is observed while the reflection from the gain side is <8%. The experimental results agree well with the analytical predictions, and prove that the orientation of the unidirectional transparency can be controlled by tuning the spacing between the loss and gain materials within half wavelength.

## Discussion

We propose and demonstrate a method to control the exceptional points of PT symmetric systems by tuning the spacing between the loss and gain materials. This enables us to shift the exceptional points to desired frequencies and control the orientation of unidirectional transparency. Our control method can be applied to different fields such as electronics, optics, microwaves and so on. Numerically, this control method can also handle PT symmetric periodic structures^9^ or non-Hermitian systems without PT symmetry^11^ (Supplementary Discussion). Thanks to the active acoustic gain medium, this control can be realized over a large frequency band. Unidirectional transparency for pulsed signals can be perceived if the transmitted phases and the non-vanishing reflections are adjusted to be identical at different frequencies using additional degrees of freedom such as number of unit cells, lengths of loss and gain units, and so on. This control ability over the exceptional points in PT symmetric system opens a new avenue for developing asymmetric wave transport devices, important for directional imaging and sensing, logic acoustics and other applications.

## Methods

### Loss material design

In this article, the acoustic refractive index is defined as *n*=343/*c*, where *c* is the complex-valued sound speed in the medium. We use a 0.148-m-long leaky waveguide consisting of nine slits with filling ratio 0.19 on the wall of the waveguide to form our loss part as shown in Fig. 2a. These slits leak the acoustic energy out of the waveguide. The height of the waveguide we use is 0.024 m. Both simulations and experiments confirm that this loss material is dispersive.

### Active gain design

An active unit containing two arrays of Kobnite 254-PS600-RO speakers facing opposite directions is used to reproduce an artificial acoustic gain material because there is no passive acoustic gain material that exists in nature. Each speaker array possesses 15 speakers with spacing 0.03 m in between the centres of each speaker. The 0.03-m spacing is chosen because it is about half of the wavelength of acoustic wave at 6 kHz, the highest frequency we used in our experiment, and hence generate plane wave in the waveguide. The two speaker arrays are used to control the transmission and reflection of the artificial gain material to generate the scattering matrix, an acoustic gain material using a linear operation described in Supplementary Note 2. A third speaker array is used to generate the incident plane wave in our experiment.

### Measurement and calibration

These three speaker arrays are controlled by a LabVIEW programme through NI-USB 6259 DAQ system. Two pairs of PUI Audio PUM-5250L-R microphones are assembled on each side of the media to measure the scattering matrix (that is, transmission and reflection coefficients). Each pair of microphones contains two microphones facing opposite directions. The measured signals are amplified and filtered using home-made amplification and filtering circuits. A calibration operation (Supplementary Note 3) is applied to the two microphone pairs so that they can detect the left and right going waves simultaneously.

## Additional information

**How to cite this article:** Shi, C. *et al.* Accessing the exceptional points of parity-time symmetric acoustics. *Nat. Commun.* 7:11110 doi: 10.1038/ncomms11110 (2016).

## Supplementary Material

Supplementary Information Supplementary Figures 1-8, Supplementary Notes 1-7, Supplementary Discussions 1-2 and Supplementary References.
