# Supplementary material for: Accessing the exceptional points of parity-time symmetric acoustics
Source: Nat Commun. 2016 Mar 30;7:11110. doi: 10.1038/ncomms11110 (PMC4820938; doi:10.1038/ncomms11110)
Supplement: Supplementary Information — Supplementary Figures 1-8, Supplementary Notes 1-7, Supplementary Discussions 1-2 and Supplementary References. [file ncomms11110-s1.pdf]

Supplementary Information

Supplementary Figures

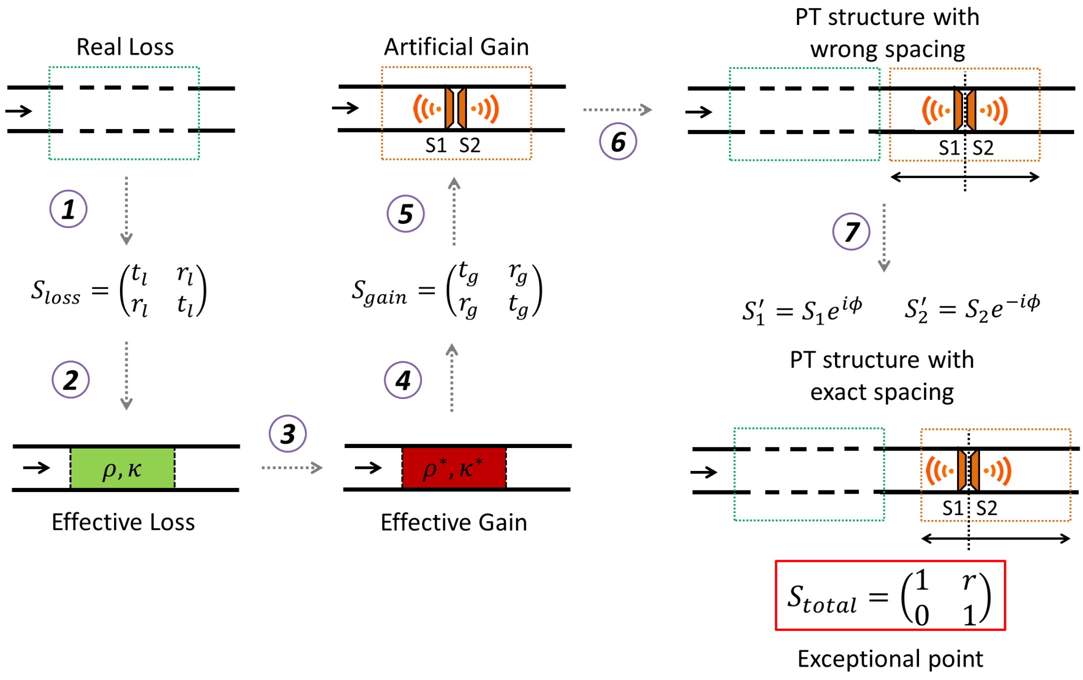

Supplementary Figure 1 | Seven experimental steps of realizing unidirectional transparency and observing exceptional points in PT symmetric systems. (1) Measuring the scattering matrix of the loss material. (2) Retrieving the effective parameters of the loss material. (3) Calculating the effective parameters of the gain material. (4) Deriving the scattering matrix of the gain material using the calculated effective parameters. (5) Tuning the active gain unit to mimic the actual gain material needed in the system. (6) Satisfying the PT symmetric condition where the loss and gain materials are precisely balanced. (7) Tuning the spacing between the loss and gain materials by apply opposite electrical phase shifts to the two active emitters to control the exceptional point and realize unidirectional transparency.

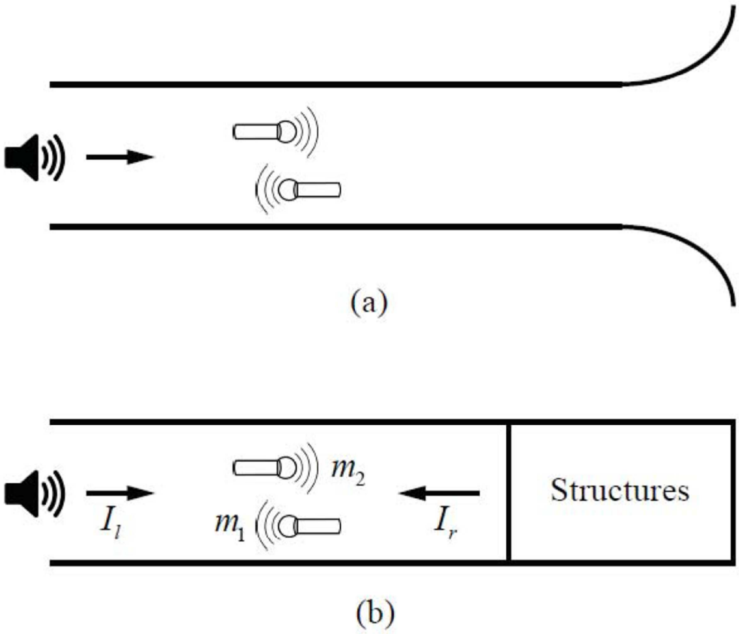

Supplementary Figure 2 | Calibration of unidirectional microphone pair. (a) Microphone pair mounted in a waveguide with anechoic ends to measure the back-front-ratio of each microphone. (b) Microphone pair assembled in a waveguide where the structures cause reflection of the incident wave, and hence left and right propagating waves exist in the waveguide.

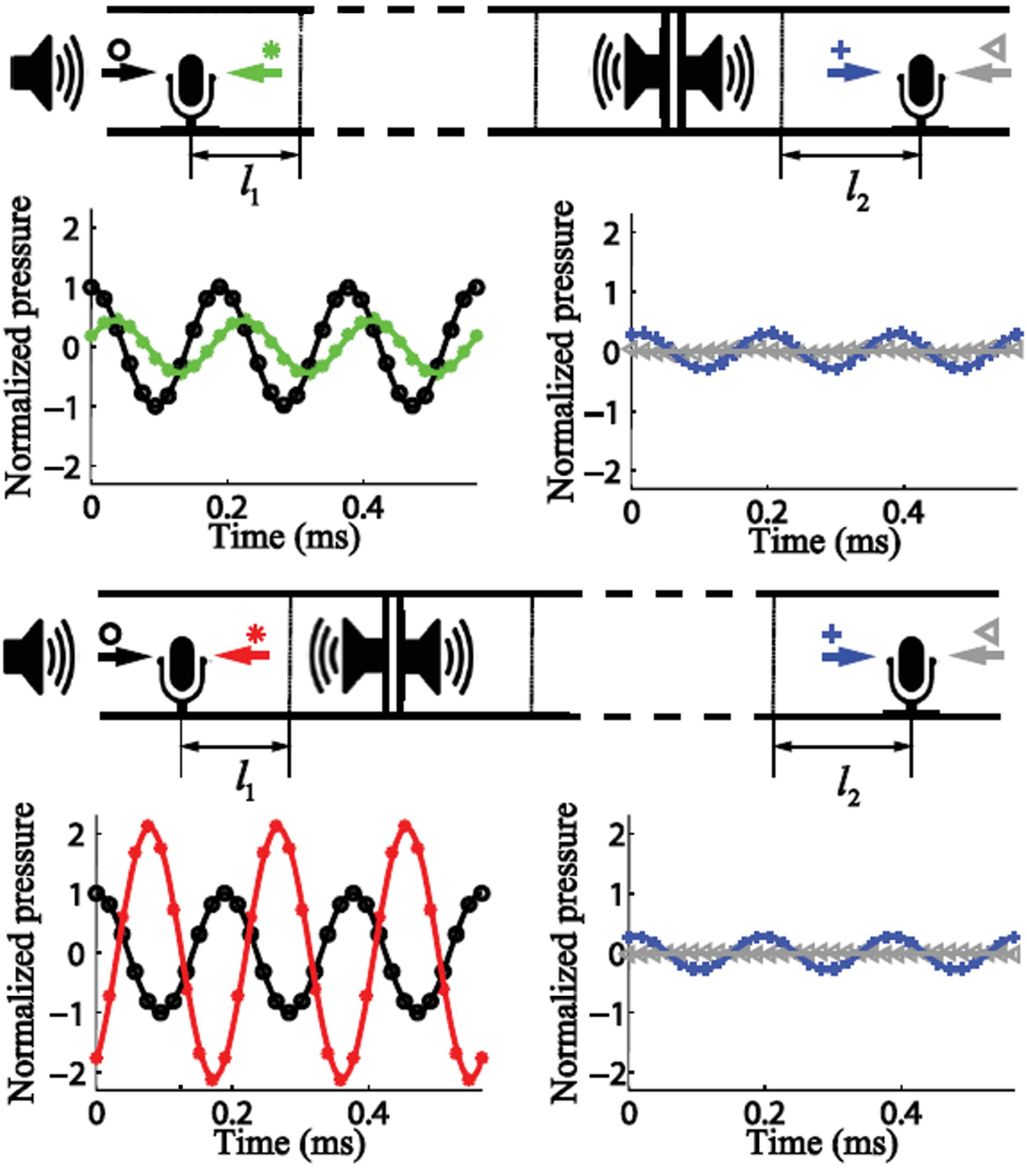

Supplementary Figure 3 | Transmission and reflections from the loss and gain sides of a PT symmetric system consisting of loss and gain units without gap in between. The calculated values are represented by curves and the measured values are represented by marked dots, respectively. Black color shows the incidence, green color shows the reflection from the loss side, red color shows the reflection from the gain side, blue color shows the transmission, and grey color shows the wave reflected from the end of the waveguide, respectively. All waves are normalized by their inputs. The system has small transmission. Reflections can be observed on both sides.

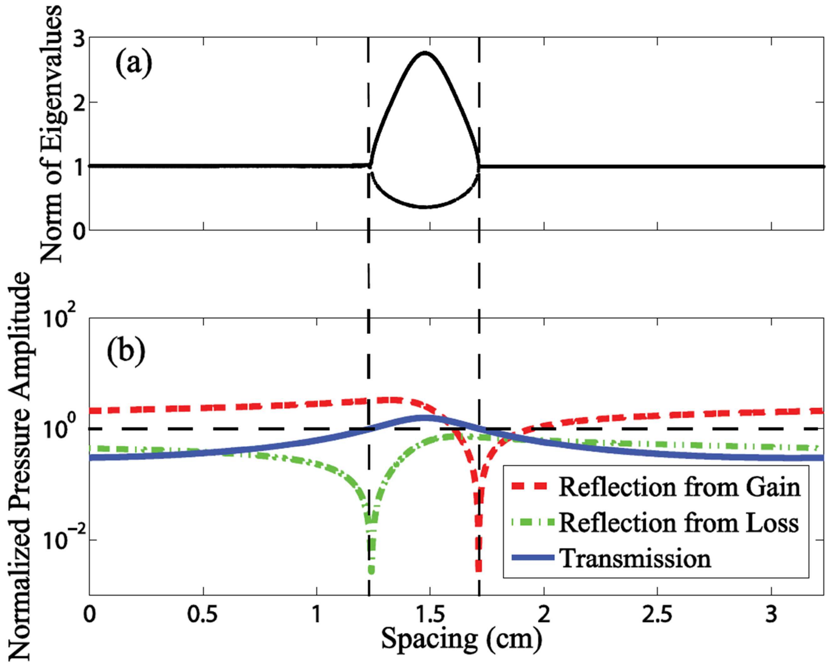

Supplementary Figure 4 | Equivalence of two definitions of PT phases. (a) The norm of the eigenvalues of the system scattering matrix varying with spacing between the loss and gain materials. (b) The transmission (blue) and reflections from the gain side (orange) and the loss side (red) varying with spacing between the loss and gain materials from zero to half wavelength at 5.3 kHz. Two exceptional points are observed when the spacing is 1.24 cm and 1.71 cm.

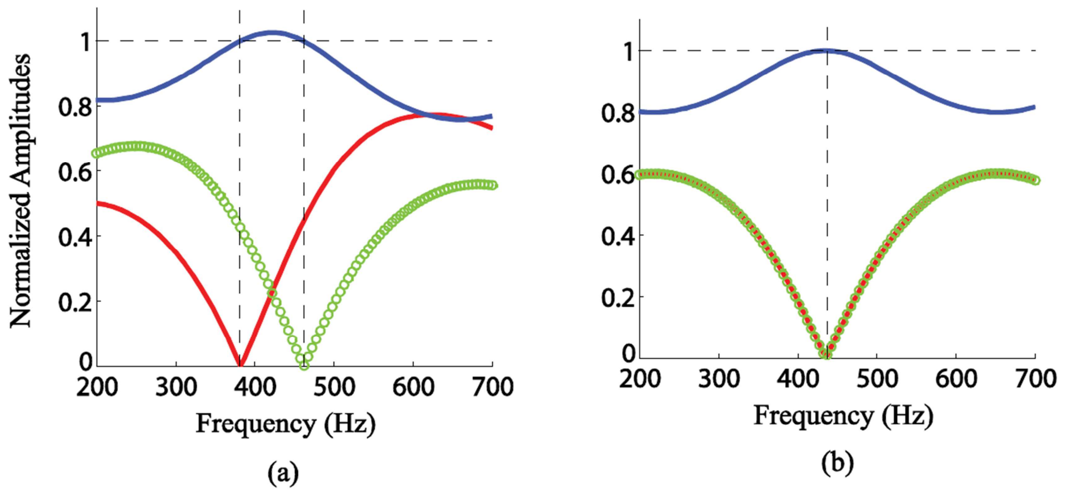

Supplementary Figure 5 | Difference between exceptional points and accidental degenerate points. Transmissions (blue curves) and reflections from the gain side (red curves) and from the loss side (green circles) of systems where (a) two exceptional points are observed and (b) one

accidental degenerate point is observed. All amplitudes are normalized by their inputs.

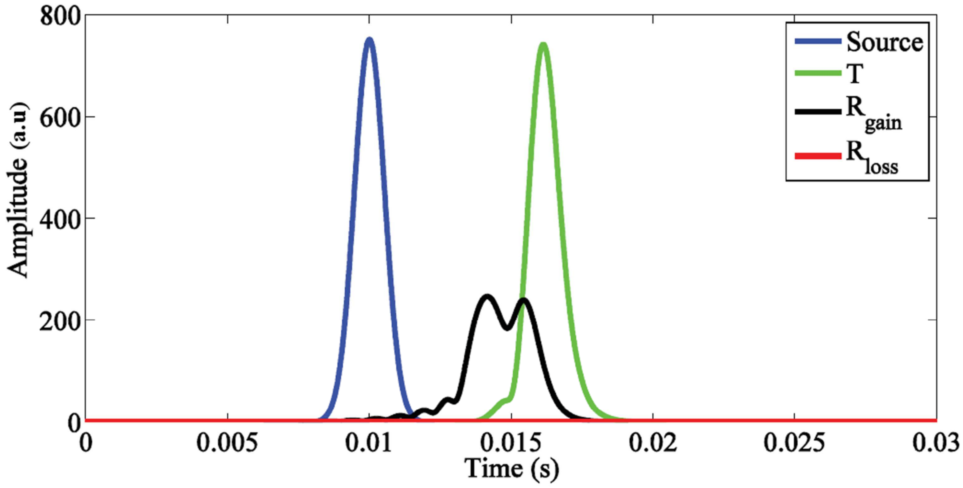

Supplementary Figure 6 | Transmission and reflections from the gain and loss sides of a pulse incidence with central frequency at 5.5 kHz and bandwidth 2 kHz. The pulse incidence, transmission, and reflections from the gain and loss sides are shown by blue, green, black, and red curves, respectively. The reflection from the loss side vanishes while the reflection from the gain side is obtained. Because the transmission phases at different frequencies are different, the transmitted pulse is slightly distorted, but the transmitted energy remains 100%.

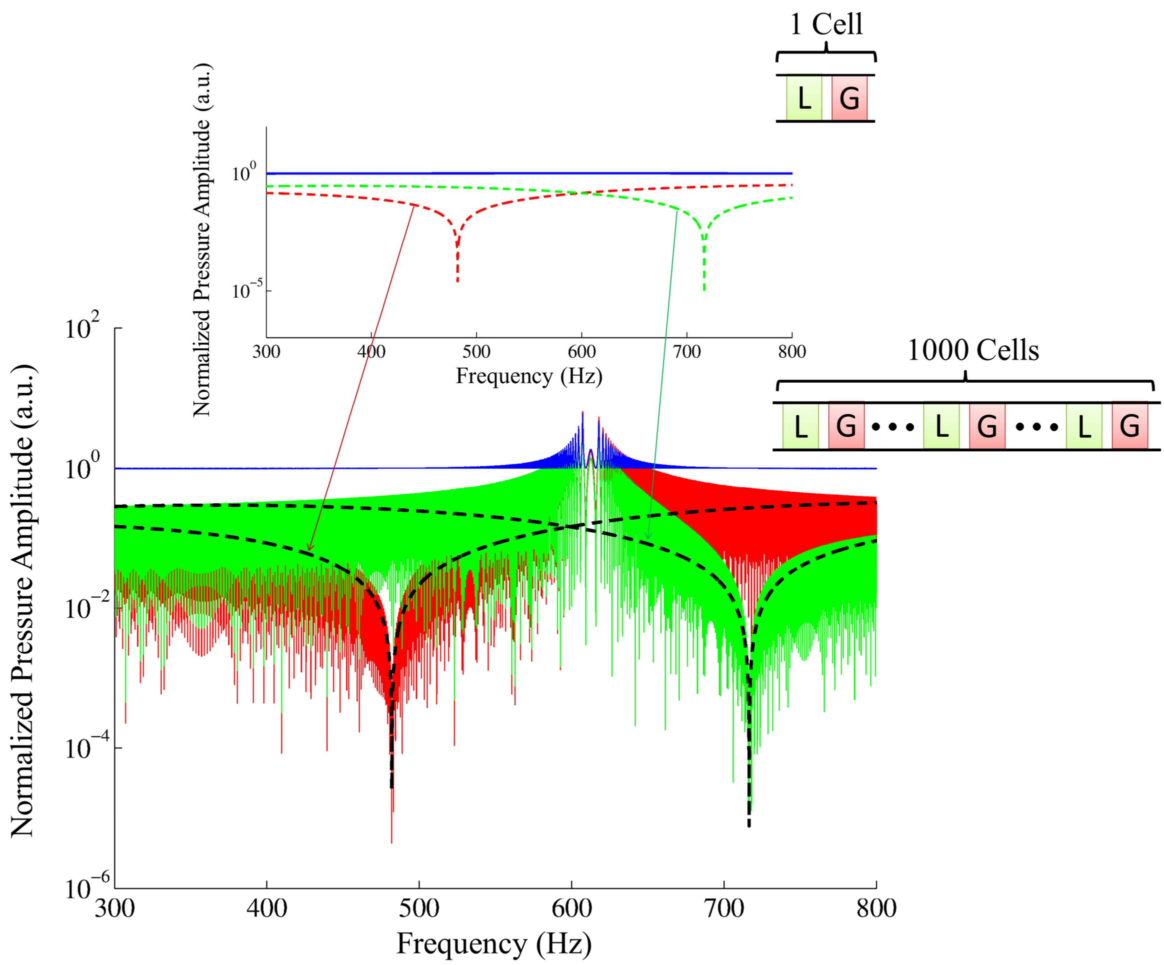

Supplementary Figure 7 | Transmissions (blue) and reflections from loss side (green) and gain side (red) of systems with one and a thousand PT symmetric units. Effective loss and gain materials with mass density 1.4 kg/m^3 and refractive indices $1.4 \pm 0.1i$ are used. In both cases, two exceptional points are observed. The exceptional points of these two systems appear at the same frequencies.

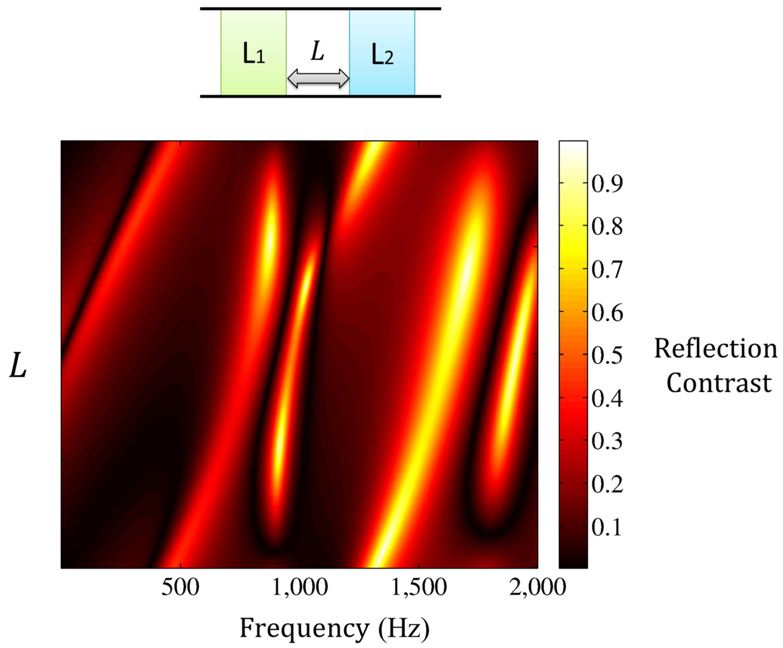

Supplementary Figure 8 | Contrast between the two reflections as a function of frequency and spacing in a system with two loss materials. Each of the loss materials is 10 cm with mass densities 1.4 kg/m^3 and 1 kg/m^3 and refractive indices $1.4-0.1i$ and $1.2-0.05i$, respectively. High contrast can be observed along several curves.

Supplementary Notes

Supplementary Note 1

Measured scattering matrices at 5.3 kHz. The measured scattering matrix of the loss unit form by the leaky waveguide is

$$S_{\text{loss}} = \begin{pmatrix} t_1 & r_1 \\ r_1 & t_1 \end{pmatrix} = \begin{pmatrix} 0.33\exp(-27^\circ i) & 0.39\exp(95^\circ i) \\ 0.39\exp(95^\circ i) & 0.33\exp(-27^\circ i) \end{pmatrix}, \quad (1)$$

with transmission coefficient t_1 and reflection coefficient r_1 of the loss part. The effective loss parameters are retrieved from this scattering matrix. The gain scattering matrix is calculated using the complex conjugate of the loss parameters

$$S_{\text{gain}} = \begin{pmatrix} t_2 & r_2 \\ r_2 & t_2 \end{pmatrix} = \begin{pmatrix} 1.49\exp(11^\circ i) & 1.75\exp(69^\circ i) \\ 1.75\exp(69^\circ i) & 1.49\exp(11^\circ i) \end{pmatrix}. \quad (2)$$

After tuning the active gain unit to present this scattering matrix and tuning the spacing between the loss and gain units to 1.24 cm, the scattering matrix of the entire PT symmetric system is measured

Considering the wave propagation between the boundaries of the PT symmetric system and the

two microphone pairs, the calculated transmission and reflection coefficients are $t = \exp(128^{\circ} i)$ and $r_g = 3.17\exp(75^{\circ} i)$. The measured amplitudes are within 5% relative error, and the measured phases are within 3% relative error.

$$S = \begin{pmatrix} t_l & r_g \\ r_l & t_g \end{pmatrix} = \begin{pmatrix} 0.99\exp(130^{\circ} i) & 3.34\exp(63^{\circ} i) \\ 0.02\exp(167^{\circ} i) & 1.02\exp(118^{\circ} i) \end{pmatrix} \quad (3)$$

Supplementary Note 2

Control of the artificial gain medium. Different from the conventional opinion that acoustic gain materials only have the function to amplify acoustic signals, actual acoustic gain media, if exist, create a certain scattering pattern whose transmission and reflection coefficients are specific and determined by the effective parameters. To mimic an actual acoustic gain material, we need to tune our active gain unit to generate the same scattering matrix. This section discusses the method to control the scattering matrix and virtual position of our active gain unit to mimic the behavior of an actual acoustic gain material that is precisely balance with the loss part at an arbitrary frequency and tuning the spacing between these loss and gain units to control and explore the exceptional points.

As presented in Supplementary Figure 1, our experiment starts with measuring the scattering matrix of the loss part form by the slits shown in Figure 2a. The loss unit is assembled in an acoustic waveguide with anechoic ends. Two calibrated unidirectional microphone pairs (discussed in Supplementary Note 3) are placed on two sides of the loss unit to measure the incidence, transmission, and reflection in the system at a chosen frequency below 6 kHz. An acoustic source formed by fifteen speakers that can generate acoustic plane waves lower than 6 kHz in the waveguide is used to generate the incident wave for the measurement. The measured transmission and reflection coefficients, i.e. scattering matrix, are used to extract the effective parameters at this frequency using the method discussed in [1]. To satisfy the PT symmetric condition, the effective parameters of the actual gain material we need to use are complex conjugates of those of the loss material (Supplementary Figure 1). Thus, the scattering matrix of the gain material is calculated using these effective parameters, and this scattering matrix is the one our artificial gain material needs to generate in our experiment. The virtual position of our artificial gain material, which tunes the spacing between the loss and gain materials, is controlled by modifying the phases of the two speaker arrays used in the gain unit. The appropriate value of the spacing that allows us to observe the exceptional point and realizes unidirectional transparency is calculated by sweeping the spacing size between zero and half wavelength using a transfer matrix method. With the appropriate spacing and the measured parameters of the loss and gain materials, the total scattering matrix of the system is calculated and used to tune the artificial gain unit in our experiment.

A linear control method is used to tune the active gain unit formed by two speaker arrays (identical to the one that generates the incident wave) that generates acoustic plane waves between 5 kHz and 6 kHz in the waveguide. The loss and gain units are assembled in a

wavelength with anechoic ends, where two pre-calibrated unidirectional microphone pairs are placed on the two sides of the unit cell formed by the loss and gain units to measure the transmission and reflection coefficients of the entire system. Because the system is linear, the detected signals of the unidirectional microphone pairs are linear combinations of the system responses to the three speaker arrays installed in the setup. Therefore, we turn only one of the speaker arrays each time and record the incidence, transmission, and reflection in the system. After we finish these measurements for the three speaker arrays, a matrix relating the emissions of the speaker arrays and the system response is generated. By inverting this measured matrix and applying the desired system response at the exceptional point, we calculate the expected emissions of these three speaker arrays. With these calculated expected speaker emissions, we tune our active gain unit to observe the unidirectional transparency of the system. In our linear system, the scattering matrix of the gain material should be the one we calculated previously when the scattering matrix of the entire system is tuned to the desired values.

Supplementary Note 3

Calibration of unidirectional microphone pairs. Unidirectional microphones that only measure acoustic waves travel in one direction are crucial for our measurement of the incidence, transmission, and reflection in the system. To realize such unidirectional microphones, we use two PUI Audio PUM-5250L-R microphones to form a microphone pair. Even though these microphones are labeled as unidirectional microphones, they still detect some of the signals incident from the back side. For the same incident wave, the amplitudes of the signals measured by the microphone when it is facing towards the incident wave (A_f) and the opposite direction are different (A_b). We define the ratio of these two amplitudes $R = A_b / A_f$ to be the back-front-ratio of the microphone. This ratio varies with frequency and is different for each microphone. A waveguide with anechoic ends is used for the calibration of the unidirectional microphone pairs (Supplementary Figure 2a). The two microphones are fixed on a thin metal strip that can be assembled in the waveguide facing opposite directions. A speaker array discussed in Section I is used to generate the incident wave for this calibration. After the microphone pair is assembled in the waveguide, we record the measured signals of the two microphones m_{11} and m_{21}. Then we rotate the microphone pair 180° and assemble it at the same position and record the two measured signals m_{12} and m_{22}. Because the waveguide has anechoic ends, only the acoustic wave generated by the speaker array travelling in one direction exists in the waveguide. Thus, the back-front-ratios of the two microphones are $R_1 = |m_{12} / m_{11}|$ and $R_2 = |m_{21} / m_{22}|$. The phases of the measured signals m_{11} and m_{21} are calibrated to be the same because the two microphones are mount at the same location where they should detect the same phase information.

When acoustic waves propagating in opposite directions exist in the waveguide (Supplementary Figure 2b), we can use the measured back-front-ratios of the two microphones to form a

unidirectional operation and extract the signals of the left and right propagating waves I_l and I_r. The detected signals of the two microphones are

$$\begin{aligned} m_1 &= I_l + R_1 I_r, \\ m_2 &= R_2 I_l + I_r. \end{aligned} \tag{4}$$

Solving Eq. (4) for the two propagating waves, one can obtain the unidirectional operation to calibrate this microphone pair

$$\begin{aligned} I_l &= \frac{m_1 - R_1 m_2}{1 - R_1 R_2}, \\ I_r &= \frac{m_2 - R_2 m_1}{1 - R_1 R_2}. \end{aligned} \tag{5}$$

Because the back-front-ratio varies with frequency for each microphone, we need to calibrate the microphone pairs for every experimental frequency.

Supplementary Note 4

Experimental result without spacing control. To demonstrate that the tuning of spacing is crucial for controlling exceptional points and realizing unidirectional transparency, we perform a control experiment at 5.3 kHz in a system where the loss and gain units are assembled side by side without spacing. The calculated and measured transmissions and reflections from the loss and gain sides are shown in Supplementary Figure 3. Only 29% transmission is observed on both sides of the system. The reflection from the loss side is 44%, and the one from the gain side is 210%. Although the two reflections are still different, the system is not unidirectional transparent. This experimental result reveals that the system is not operating at the exceptional points. Unidirectional transparency cannot be observed without tuning the spacing.

Supplementary Note 5

Difference between exceptional points and accidental degenerate points. Conventionally, two equivalent definitions exist for PT exact and broken phases. A PT symmetric system is in PT exact phase when the eigenvalues of the system scattering matrix are unimodular as shown in Supplementary Figure 4a. Once the norms of these eigenvalues are non-unity, the system is in the PT broken phase. As depicted by Supplementary Figure 4a and Supplementary Figure 4b, this definition of the PT phases is equivalent to the one we gave in the main text.

As defined previously, exceptional points of PT symmetric systems associate with the phase transition of the system where total transmission is observed. According to energy conservation, one of the reflections must vanish at the exceptional points. Total transmission can also be obtained for accidental degenerate points where the two reflections are simultaneously zero. The exceptional points and accidental degenerate points look similar, but they are fundamentally different. Supplementary Figure 5 shows the transmissions and reflections for two PT symmetric systems. Two exceptional points associated with the phase transitions from the PT exact phase to PT broken phase, and back to PT exact phase are observed in Supplementary Figure 5a. By

tuning the system parameters, one can shift these two exceptional points and make them to be accidentally degenerate at a frequency, resulting in an accidental degenerate point shown in Supplementary Figure 5b. However, the PT broken phase does not exist when this accidental degenerate point appears. Thus, the phase transition does not occur at accident degenerate points, making them different from the exceptional points.

Supplementary Note 6

Broadband pulse signal excitation. A pulse signal with central frequency at 5.5 kHz and bandwidth 1 kHz is used to study the response of broadband signal in the PT symmetric structure under our control of the exceptional points. The calculated transmission, reflections from the loss and gain sides are shown in Supplementary Figure 6. The reflection from the loss side is zero, while the reflection from the gain side is observed. Because the phases of transmission at different frequencies are different, the transmission is slightly distorted, but the transmitted energy remains 100%. This analytical result reveals that our control of the exceptional points is working for broadband signals.

Supplementary Note 7

Exceptional points of PT symmetric periodic structures. Even though only one PT symmetric unit is used in our demonstration, the method we used to control exceptional points can be used for PT symmetric periodic structures described in [2]. Supplementary Figure 7 shows the comparison of transmission and reflection loci of PT symmetric systems consisting of one unit and a thousand units. Effective loss and gain media with mass density 1.4 kg/m^3 and refractive indices $1.4 \pm 0.1i$ are used in the simulations. Each of the loss or gain unit is 10 cm with no spacing between them. Two exceptional points are observed at 482 Hz and 716 Hz in both cases, indicating that the exceptional points of systems with single and periodic PT symmetric units are identical. This numerical result indicates that our control method valid for one PT symmetric unit can also be used to control the exceptional points of systems with periodic PT symmetric units regardless of the number of unit cells.

Supplementary Discussion

Control of exceptional points in systems formed by multiple non-Hermitian materials without PT symmetry. Our control method of exceptional points can also handle systems containing multiple non-Hermitian materials without PT symmetry discussed in [3] for the observation of unidirectional reflection. Supplementary Figure 8 shows the contrast of reflections from the two sides of the system containing two 10 cm effective loss materials with mass densities 1.4 kg/m^3 and 1 kg/m^3 and refractive indices $1.4-0.1i$ and $1.2-0.05i$, where the contrast is defined as

$$\text{Contrast} = \frac{\left||r_{\text{left}}| - |r_{\text{right}}|\right|}{|r_{\text{left}}| + |r_{\text{right}}|}. \qquad (6)$$

This contrast is a function of frequency and the spacing between the two loss materials. Several curves with high contrast are observed in Supplementary Figure 8. One can tune the spacing according to the incident frequency following these curves to control the exceptional points and realize unidirectional reflection with high contrast.

Discussion on previous demonstration of PT symmetric acoustics. A recent report discussed the possibility of using electronic circuits with specific impedances to realize a PT symmetric acoustic unit [4]. However, only one exceptional point was observed, and no method for the control of this exceptional point was proposed. This realization of PT symmetric system relies on electronic signals that has been demonstrated previously [5], rather than acoustic waves. Even with an active gain unit, the validation of the PT symmetric condition in our system depends on the acoustic waves propagating through the loss and gain units. Both loss and gain units need to be judiciously designed in their proposal, preventing a wide application because most natural materials contain loss that is difficult to be controlled. Our design of PT symmetric acoustic systems overcomes this limitation because only the active gain unit is tuned to match with arbitrary loss materials.

Supplementary References

[1] Fokin, V., Ambati, M., Sun, C., and Zhang, X., Method for retrieving effective properties of locally resonant acoustic metamaterials, *Phys. Rev. B* **76**, 14432 (2007).

[2] Lin, Z., Ramezani, H., Eichelkraut, T., Kottos, T., Cao, H., and Christodoulides, D. N., Unidirectional invisibility induced by PT-symmetric periodic structures, *Phys. Rev. Lett.* **106**, 213901 (2011).

[3] Feng, L., Xu, Y. L., Fegadolli, W. S., Lu, M. H., Oliveira, J. E. B., Almeida, V. R., Chen, Y. F., and Scherer, A., Experimental demonstration of a unidirectional reflectionless parity-time metamaterial at optical frequencies, *Nature Mater.* **12**, 108 - 113 (2013).

[4] Fleury, R., Sounas, D., and Alu, A., An invisible acoustic sensor based on parity-time symmetry, *Nature Comm.* **6**, 5905 (2014).

[5] Schindler, J., Li, A., Zheng, M. C., Ellis, F. M., and Kottos, T., Experimental study of active LRC circuits with PT symmetries, *Phys. Rev. A* **84**, 040101 (2011).
